# Effects of workplace bullying on Chinese children’s health, behaviours and school adjustment via parenting: study protocol for a longitudinal study

**DOI:** 10.1186/s12889-019-6458-1

**Published:** 2019-01-30

**Authors:** Catalina Sau Man Ng

**Affiliations:** Department of Early Childhood Education, Education University of Hong Kong, 10 Lo Ping Road, Tai Po, New Territories Hong Kong

**Keywords:** Workplace bullying, Longitudinal study, Chinese children, Parenting, Child well-being, Externalizing behaviors

## Abstract

**Background:**

Bullying in the workplace is a serious public health issue. As a chronic work stress, workplace bullying places the victims’ physical and mental health at risk which, in extreme cases, may lead to suicidal ideation. The high prevalence rate of workplace bullying has been reported and documented globally. However, a major limitation of the existing literature is that studies essentially focus on the consequences of workplace bullying on victims, including the psychological, physiological and socioeconomic impacts, and on the factors causing workplace bullying, but research on the impact of workplace bullying on the victims’ families is lacking. It is however evident that the consequences of workplace bullying have a spillover effect on the victims’ family members. Since many victims have children and given that children are particularly vulnerable to a negative family environment, examining the impacts of the type of parental stress induced by workplace bullying on children’s health including physical and psychological health (depression and self-esteem), externalizing problem behaviors (aggression, lying, disrespect) and school adjustment (academic performance and school conduct) is urgently needed. The overall aim of this study is to examine how health, externalizing problem behaviors and school adjustment of children whose parents are victims of workplace bullying may be associated with the impact that workplace bullying has on parenting practices.

**Methods:**

It is a longitudinal study. Quantitative data will be collected from multi-informants, including currently employed Chinese parents, their children aged between 6 and 12 years old, and their class teachers at two time points, separated by a 1-year interval. One primary school will be recruited from each district of Hong Kong including participants with different socioeconomic backgrounds. At least 837 dyads (parents and children) from 18 primary schools will join the study.

**Discussion:**

Workplace bullying not only affects victims but can also be harmful to their families as it alters the victims temper in the family environment. Results will be informative for the government and corporations to make concerted efforts and find strategies to prevent workplace bullying and to heighten the awareness of the importance of promoting safe and respectful workplaces for workers.

## Background

The World Health Organization (WHO) recognizes bullying in the workplace as a serious public health issue [1]. Empirical research has documented bullying-related morbidity and mortality of the victims e.g., [2–4] and the existing literature enlightens us about this phenomenon with some very disturbing facts. For example, according to the literature, the rate of severely bullied workers who reported having suicidal ideation was 6 times higher than never bullied workers [5]. In their systematic review, Leach, Poyser, and Butterworth [6] reported that there was a significant positive relationship between workplace bullying and suicidal ideation. Kivimäki et al. [7] found that there was a strong relationship between workplace bullying, depression and cardiovascular diseases. In a 3-wave prospective follow-up study, Bonde and colleagues [8] found that poor self-rated health and depression among victims persisted for up to 4 years regardless of whether the bullying had ended or not. A recent study by Xu and colleagues [9] found that workers who have been exposed to bullying or violence in the workplace had a higher risk of type 2 diabetes. So, it is evident that persistent negative interactions with bullies in the workplace can lead to chronic stress among victims which results in higher levels of anxiety, depression, health-related problems and suicidal thoughts.

The high prevalence rate of workplace bullying in Western countries reveals that it is becoming a serious problem worldwide (see Table 1). However, given the substantial prevalence of workplace bullying in the West, our information about the extent of bullying in the workplace in Asian countries is limited due to the dearth of studies in this area [10]. In Korea, Yoo and Lee [10] found that 87% of employees reported having experienced some form of bullying in the past 6 months. In Japan, according to the Workplace Power Harassment Survey (employee survey), 25.3% of all respondents reported that they experienced workplace bullying in the past 3 years and males reported more bullying in the workplace than females (26.5% vs. 23.9%) [11]. In Hong Kong, the only available data are the results of a telephone survey conducted in 2013 by an employer service consultancy on 509 Hong Kong respondents aged between 21 and 60 who were employed at the time of the survey. The results showed that about 53% of the respondents had been victims of at least one type of workplace bullying and, among the respondents who had been victims of workplace bullying, more than 68% said they had been bullied by their supervisors [12]. Information about the extent of workplace bullying is important because it can inform government policy and heighten the awareness of corporations about this phenomenon. Hong Kong is notorious for having the longest working hours among workers out of 71 cities surveyed worldwide [13], so it must be noted that long working hours combined with the chronic stress caused by workplace bullying will put workers’ physical and mental health at risk. It is therefore urgent to provide a clear assessment of the prevalence rate of workplace bullying in Hong Kong.

**Table 1:** Prevalence rates of workplace bullying in different countries [52] (p.33)

Year	First author	Country	Occupations	Sample size	Prevalence rate (%)
2001	Voss [53]	Sweden	workers in postal service	3470	3.3
2004	Varhama [54]	Finland	municipal employees	1961	16.0
2007	Niedhammer [55]	France	various workers	7694	10.2 (past year)
2007	Matthiesen [56]	Norway	various workers	2215	8.3
2009	Ortega [57]	Denmark	various workers	3429	8.3 (past year)
2011	Giorgi [58]	Italy	various workers	3112	15.2
2011	Glaso [59]	Norway	bus drivers	1023	11 (past 6 months)
2011	Lallukka [60]	Finland	various city workers	7332	5 (current workplace)
2011	Notelaers [61]	Belgium	various workers	8985	8.3
2012	Perbellini [62]	Italy	workers	449	30.1
2012	Keuskamp [63]	Australia	various workers	1145	15.2
2012	Niedhammer [64]	France	various workers	29,680	6.4
2012	Cunniff [65]	South Africa	various workers	13,911	35.1

Our knowledge on workplace bullying is limited to the empirical studies conducted in the West, which are largely confined to the victims. This suggests that the effects of workplace bullying are only harmful to the victims, not their family, or at the very least this crucial point has been overlooked [14] and some reports clearly provide support to this hypothesis. For example, on a survey conducted by Family Lives in the U.K., more than 80% of the participants reported that workplace bullying affected their family life [15]. Therefore, the “spillover” effect of workplace bullying on the victims’ personal lives [16] cannot be ignored and constitutes an important consequence of workplace bullying which has been largely neglected by the current literature. Negative job-related feelings such as anger, frustration, despair, and hopelessness can compromise parenting abilities in such a way that the stress resulting from workplace bullying may indirectly affect their children’s psychological well-being and behavior by altering their parenting practices [17]. Children are vulnerable to poor parenting as it has been shown that parenting is associated with many problems in children such as delinquency and academic problems [18]. Given that parents play a significant role in child development and since many victims of workplace bullying are also parents, it would be useful from a theoretical and practical perspective to explore how parents who are victims of workplace bullying affect child development via parenting practices.

Lee and Tiedens [19] found that interdependence strengthens the relationship between workplace ostracism and its consequences. With Chinese culture placing much emphasis on collectivism and interpersonal relationships, the impact of workplace bullying may be even more detrimental to the well-being of the victims. To my knowledge, no study has been conducted to investigate the relationship between parents being bullied in the workplace and the impacts on family members in Asia. The results can therefore serve as a wake-up call to many different parties such as parents, teachers, corporations, and government to stop workplace bullying or reduce its incidence so that children who would otherwise have to suffer the consequences of workplace bullying directed at their parents can develop and thrive in a better, happier and less stressful environment.

With a labor force of 3.88 million people which represents about half of the local population in Hong Kong [20] and given the high reported prevalence rates of workplace bullying in Western countries as a reference, we cannot under-estimate the extent and severity of the problem on both the victims of workplace bullying and the affected family members. We need to examine how workplace bullying directed at parents will ultimately affect child development so that tailor-made, culturally suitable interventions can be designed to assist the victims.

### Workplace bullying and parenting

Workplace bullying causes high levels of stress in victims over a prolonged period of time and chronic stress has been found to be associated with adverse family functioning [17] and parent-child dyads [21]. In particular, the increased stress, anger and anxiety resulting from work stress may change the communication patterns with family members. There is less positive interaction with spouse and children because victims are preoccupied by the bullying experience and by the need to talk about the bullying experience. The victims are often withdrawn at home, so they will be perceived as inaccessible and disengaged [22].

The overwhelming negative feelings brought about by workplace bullying may cause the victims to have more conflicts with their spouse over even trivial matters due to mood swings. Duffy and Sperry [23] highlighted that workplace bullying has a significant impact on marriage as adverse feelings from workplace bullying lead to an increase in marital tension. Children living in a family with frequent conflicts also exhibit more problems in terms of social, emotional, and behavioral functioning [24]. This idea is also supported by several Western studies investigating the possible impact of work stressors on parenting, which found that parents who are exposed to chronic job stress appear to be more controlling with their children so that the parent-child relationships are characterized by less cohesion and more conflict [25].

### Theoretical framework: Ecological systems theory

The proposed study will adopt Bronfenbrenner’s ecological systems theory [26] as a theoretical framework to understand how the environment affects children development. Based on Bronfenbrenner’s original theory, the context has four distinct concentric systems (micro, meso, exo, and macro), and each system can have a direct or indirect impact on a child’s development. A fifth system (chrono) was later included in the model. A brief illustration of Bronfenbrenner’s ecological systems theory relevant to this study is outlined below.

The microsystem is the first level or innermost layer of Bronfenbrenner’s ecological model. It is the most immediate environment including groups and institutions which the child has direct interactions with. Children’s microsystems include immediate relationships (family, playmates) or organizations (schools) where the proximal processes occur [27]. The relationships at this level can be bidirectional as the family of the child can influence the child’s behavior and his/her behavior can influence the family. The second immediate level is the mesosystem. It connects two or more different microsystems such as home and school. For instance, what happens in a microsystem, such as the home in which a child lives, can have an impact on the way the child behaves at school, and what happens at school can also affect the child’s behavior at home. The exosystem constitutes the third layer and children do not have direct interactions at this level. However, the system still has an indirect impact on a child’s development because it contains the micro and mesosystems. Thus, if the wellbeing of the people who have direct contact with children is affected, this may have consequences for the wellbeing of those children too. For example, a parent’s workplace schedule such as shift work may prevent a parent from coming home early enough to see their child before bedtime, depriving the child of parental care. By applying the ecological systems theory into the present study, it is hypothesized that parents who are victims of workplace bullying affect the child’s development through their parenting, which matches with the concept of exosystem and with the idea that, although children may not be the direct victims of workplace bullying, they may be indirect victims due to the poor parenting practices of parents resulting from workplace bullying. Therefore, if the negative emotions induced by workplace bullying affect parental behavior and parenting practices towards children, this may ultimately cause children to suffer and become indirect victims of workplace bullying.

#### Study objectives

**Fig. 1:**
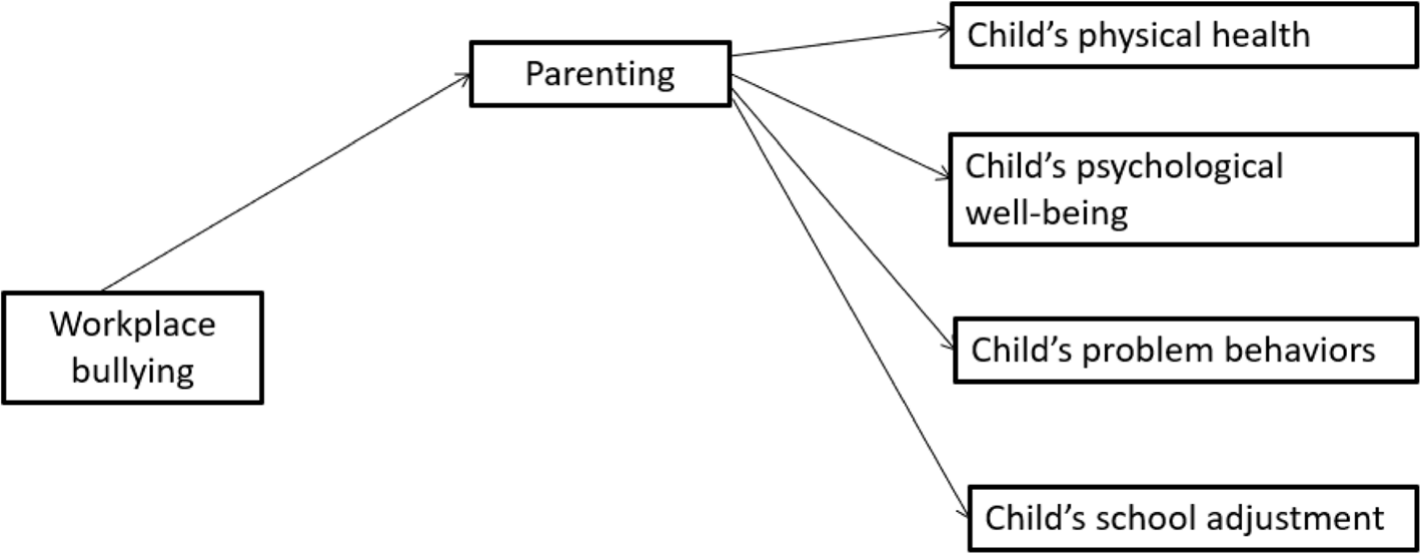
A hypothesized model of the effects of workplace bullying and parenting on the child’s health, psychological well-being, problem behaviors and school adjustment

The objectives of the study are:

1. To estimate the annual and lifetime prevalence of workplace bullying in Hong Kong;
2. To test in a hypothesized model the mediating role of parenting on the relationship between parents who are victims of workplace bullying and child’s health, psychological well-being, behaviors and school adjustment (see Fig. 1);
3. To explore whether parents being bullied at baseline is predictive of any explanatory variables (children’s health, psychological well-being, problem behaviors and school adjustment) at follow-up.

## Methods

### Study design

The study is a longitudinal survey. Two waves of data collection will be separated by a 1-year interval. In order to examine the cause and effect relationship between parents who are victims of workplace bullying at baseline and the potentially adverse impact on their child’s well-being at follow-up via poor parenting, a longitudinal design is needed.

### Definition of workplace bullying

A definition is needed to measure the problem of workplace bullying. However, there is no universally agreed-upon definition for workplace bullying. Based on an exhaustive literature review, the operational definitions of workplace bullying often consist of five characterizing elements which are, “(1) victims experience negative behavior; (2) behaviors are experienced persistently; (3) victims experience some harm, either psychological or physical; (4) victims perceive they have less power than the bully and, thus have difficulty defending themselves; and (5) victims label themselves “bullied” [28] (p. 273) and other definitional criteria, e.g., intent and behavior that violated a standard of appropriate workplace behavior” [29] (p. 347). The definition formulated by Einarsen, Hoel, Zapf, and Cooper [30] has been commonly used in the West and using the same definition will therefore enable the comparison of findings from the present study with existing research conducted in the West. Einarsen, Hoel, Zapf, and Cooper’s [30] definition states that “bullying at work means harassing, offending, socially excluding someone or negatively affecting someone’s work tasks. In order for the label bullying (or mobbing) to be applied to a particular activity, interaction or process it has to occur repeatedly and regularly (e.g., weekly) and over a period of time (e.g., about six months). Bullying is an escalated process in the course of which the person confronted ends up in an inferior position and becomes the target of systematic negative social act” (p. 15).

### Sample size and statistical power

Based on a meta-analysis of the prevalence rates of workplace bullying, 11% of the workers in the population experienced workplace bullying [31]. Using a smaller margin of error of 2.5% to guarantee that the correct number of bullied parents will be sampled, and a 95% selected confidence interval, the required sample size should include 585 participants. Assuming a 90% sample acceptance rate, the sample required should increase to 669 participants. However, for longitudinal studies, the longer the follow-up period, the higher the drop-out rate [32] with attrition rates ranging from 30 to 70% being often reported [33]. Since this study is a school-based study, the attrition rate is estimated to be approximately 20% after a year for the longitudinal study based on previous studies on schoolchildren [34, 35], therefore a sample size of at least 837 dyads (parents and children) is required.

### Participants

The inclusion criteria for parents, teachers and students are listed below:

1. Parents: (i) Chinese; (ii) aged 18 or above; (iii) currently working either full-time or part-time; and (iv) having at least one child studying in Primary One to Primary Five.
2. Teachers: Class teachers of Primary One to Primary Five will be invited to join. If class teachers decline, subject teachers teaching the same classes as class teachers will be invited. Teachers teaching major subjects (Chinese, English and Mathematics) will first be invited, followed by teachers teaching other subjects. This is because teachers of major subjects give lessons every day and they know students better.
3. Children: Chinese students studying Primary One to Primary Five at selected schools will be invited to join the study, with the exclusion of those with cognitive and learning problems.

The exclusion criteria are the following:

1. Parents: stay-at-home mothers and fathers
2. Children: Students of Primary Six are excluded because they will already have left primary school at the time of the follow-up data collection.

### Recruitment of participants

The participants of this study are Chinese parents who are currently working full-time or part-time, class teachers and Primary One to Primary Five students studying in local primary schools from 18 districts in Hong Kong. Primary schools will be randomly selected from the school lists by district posted on the website of the Education Bureau (http://www.edb.gov.hk/en/student-parents/sch-info/sch-search/schlist-by-district/school-list-cw.html). International primary and special needs schools will be excluded. Emails will be sent to the randomly selected schools on the school lists. If principals from any schools are not replying they will be contacted by phone a week later. If the school declines to join the study, another school randomly selected from the list in the same district will be invited. One primary school from each of the 18 districts of Hong Kong will be invited to join the study so as to cover the entire Hong Kong territory and increase the representativeness. Ethics approval from the Human Research Ethics Committee at the corresponding author’s university has been sought. Written consent from all the participants will be obtained prior to data collection.

### Data collection

#### Procedures of data collection at baseline – Parents

After the schools agree to join the study, letters of invitation to the parents of primary students studying Primary One to Primary Five in those schools will be sent over. Parents will be required to respond to the invitation by accepting or refusing the invitation. Collecting both yes and no reply forms is a useful measure to ensure that all parents were effectively given the letter of invitation by their children. All consenting parents will then receive the questionnaire and instructions for completing the questionnaire in a sealed envelope via their children. Each parent will complete the questionnaire independently and the completed questionnaire will be placed in a sealed envelope which will be provided along with the questionnaire. The completed questionnaire will be returned by the children to the class teachers on the specified date, who will then hand them over to the PI. Participating parents will use their child’s unique Student Reference Number (STRN) provided by the Education Bureau in order to keep their identity anonymous and to match individual data correctly as the follow-up data will be collected after a 1-year interval.

#### Procedures of data collection at baseline – Teachers

The consenting class teachers of the participating schools will complete questionnaires for students who agreed to join the study. The class teachers will be given a list of the students’ class number so that they can rate their behaviors and academic performance. Once completed, each class teacher will place the completed questionnaires and the list in a large sealed envelope provided for collection.

#### Procedures of data collection at baseline – Students

Primary One to Primary Five students will be encouraged to complete the questionnaire in class or at home depending on the school arrangement. Since primary students may not be able to understand the questions in the questionnaire, special arrangement will be made. In each classroom, the questions will be read aloud by the PI or a trained research assistant while students rate each item. Some trained research assistants will also be there to help monitor the students’ progress throughout the completion of the questionnaires. They will answer questions about individual items, re-read questions, and make sure that students complete the questionnaires at a similar pace. Adequate time will be given for completing the questionnaires. This data collection procedure is commonly used in many local studies with young students [36].

#### Follow-up (1-year interval)

The same procedure will be used for the follow-up data collection with parents, teachers and children.

### Outcome measures

The key variables in the hypothesized model (see Fig. 1) were proposed because workplace bullying has a severe impact on the psychological well-being of bullied individuals. Children whose parents are stressed are at a greater risk of developing depressive symptoms. Stressed parents are less supportive and more neglectful of children, which in turn affects children’s health and causes more internalizing and externalizing problems [37]. Some externalizing problems are exhibited in common problematic behaviors among children such as aggression, lying, and disrespect towards adults. In addition, since bullied parents are likely to be less involved in their children’s academic studies, children’s academic performance may also be affected, which in turn has an impact on children’s self-esteem.

### Primary outcomes

The primary outcomes of this study are psychological well-being, physical health, externalizing problem behavior and academic adjustment.

1. Psychological well-being

Students completed the following scales so their psychological well-being could be assessed:

(i) Center for Epidemiology Studies – Depression Scale (CES-D) (Chinese version);
(ii) Rosenberg’s Self-Esteem Scale (Chinese version).

(i) *Child depression*. It will be measured by the 20-item Chinese version of the Center for Epidemiology Studies – Depression Scale (CES-D) [38]. The 20 items are rated on a 4-point Likert scale, ranging from 0 “Rarely or none of the time” to 3 “Most or all of the time”, such as “I felt depressed”, “I had trouble keeping my mind on what I was doing”, with total scores ranging from 0 to 60 and a higher score indicating a higher level of depression. A total score of 15 or above indicates a risk of depression. The scale has been translated into Chinese, yielding good internal consistency and concurrent and construct validity [39].

(ii) *Self-esteem*. Child self-esteem will be assessed by the Chinese version of the Rosenberg’s Self-Esteem Scale (RSE) [40]. The scale contains 10 items to measure global self-esteem such as “I take a positive attitude toward myself” on a 4-point Likert scale (1 = strongly disagree; 4 = strongly agree). Higher scores show higher level of self-esteem. The scale has good psychometric properties and the validity of RSE has been well established among the Chinese samples [41].

(2) Physical health

(i) *Child’s physical health*. Three single-item measures of physical health will be used, using a 4-point Likert scale ranging from “Never” (0) to “Often” (3): (i) a subjective indicator (“How would you rate your child’s physical health since his/her birth in comparison with other children?”), (ii) “In the past 6 months, how would you rate your child’s health in comparison with other children?” and (iii) "In the past 6 months, how often did your child have the following health problems? (a) hard to fall asleep, (b) abdominal pain, (c) stomachache, (d) fever, (e) cough, (f) running nose / flu.

(3) *Child externalizing problem behavior.* Twenty self-constructed items will be used to measure children’s externalizing problem behavior rated by parents using reference to Child Behavior Checklist [42] and based on the interviews with parents in the focus groups prior to the construction of the questionnaire for the Chinese context. Items are rated on a 4-point Likert scale, ranging from 0 “never” to 3 “very often”. This scale comprises typical externalizing problem behaviors for children such as “lies”, “vandalism”, “fighting”, “addiction to online gaming”. Both parents and teachers will rate the external problems of children as they may behave differently at home and at school. Students will also ask to rate whether they are satisfied with their conduct in a 5-point Likert scale ranging from “very poor” (1) to “very good” (5).

(4) *Academic adjustment*. Teachers will be asked to provide information regarding (i) the student’s position in class and the whole form in the last academic year and the current year (Primary One students will use the results in the first semester); (ii) examination scores for three major subjects (Chinese, English, and Mathematics) will be obtained for the previous year and the current year. Students will also be asked whether they are satisfied with their results in Chinese, English, Mathematics and their overall academic performance.

### Secondary outcomes

(i) *Annual and lifetime prevalence of workplace bullying.* Two single-item measures of workplace bullying will be completed by working parents. For the annual prevalence, an item such as “Have you ever been bullied at work over the last 12 months?” (Options: Never bullied/Yes, rarely/Yes, sometimes/Yes, Often). For lifetime prevalence of workplace bullying, an item such as “Have you ever been bullied at work in your life?” (Options: Never bullied/ Yes, rarely/Yes, sometimes/Yes, Often).
(ii) *Workplace bullying.* The Chinese Workplace Bullying Scale (CWBS) [43] with some adaptation will be used to measure bullying behaviors in the workplace in the local context (e.g., “I am assigned work with heavy loads.”, “Someone withholds information deliberately to hinder my work performance”). Items are changed to rate on a 4-point Likert scale, ranging from 0 “never” to 4 “Often”. Based on the interview with the participants for designing the questionnaires, more items such as “My current work exceeds the scope of the contract”, “My efforts at work have been ignored” have been included in the original scale to suit the local context. The original scale has showed good validity and reliability [43].
(iii) *Mental health.* The General Health Questionnaire-12 (GHQ-12; Chinese version) [44] contains 12 items and will be used to measure the severity of mental problems of parents over the past 4 weeks using a 4-point scale (from 0 “less than usual” to 3 “much more than usual”). Items 1, 3, 4, 7, and 12 were scored inversely and the total scores range from 0 to 36, with higher scores showing poor mental health. The scale has proven to be valid and reliable e.g., [45, 46].
(iv) *Life satisfaction*. The Satisfaction with Life Scale (SWLS-Chinese version) [47] will be used to measure the working parents’ perceptions about life satisfaction. It consists of five items which assess an individual’s global cognitive judgment of their life satisfaction (e.g., “I am very satisfied with my living conditions”. Items are rated on a 7-point scale, ranging from 1 “strongly disagree” to 7 “strongly agree”. A composite score will be calculated to obtain the mean of the life satisfaction. Adequate reliability has been reported [48].
(v) *Parenting.* Five items to assess students’ perceptions of parenting (ranging from 1 “never” to 4 “often”). Examples of which are: “In the last six months, how often did your father scold you because of his work stress?”; “In the last six months, how often did your father punish you physically because of his work stress?”.

The details of the scales for measuring the primary and secondary outcomes are summarized in Table 2.

**Table 2:** Measurements used for measuring primary and secondary outcomes

Outcome measures	MeasurementScale	Details of the measurement	Completed by		
			Parents	Teachers	Students
Primary outcomes					
1. Child depression	Center for Epidemiologic Studies Depression Scale for Children [38]	• To assess the level of depression among children/adolescents; • 20 items; 4-point Likert scale ranging from “Not at all” (0) to “A lot” (3), with higher scores indicating greater level of depressive symptoms.			X
2 Child self-esteem	Rosenberg Self-Esteem Scale [40]	• To measure students’ self-esteem; • 10 items; 4-point Likert scale ranging from “Strongly agree” (1) to “Strongly disagree” (4), with higher scores indicating higher self-esteem.			X
3. Child’s physical health	Items created by the author	• To measure students’ physical health • (i) a subjective indicator (“How would you rate your child’s physical health since his/her birth in comparison with other children?”), (ii) “In the past 6 months, how would you rate you child’s health in comparison with other children?” (iii) “Over the past 6 months, how often has your child had the following health problems? (a) hard to fall asleep, (b) abdominal pain, (c) stomachache, (d) fever, (e) cough, (f) running nose / flu. • 3 items; 4-point Likert scale ranging from “Never” (0) to “Often” (3)	X		
4. Externalizing problem behavior	Items created by the author	• To measure children’s externalizing problem behaviour • 4-point Likert scale, ranging from “Not true” (0) to “Very often” (3)	X	X	X
5. Academic adjustment	Items created by the author	• To measure the academic performance of students • (i) the student’s position in class and all classes within the students’ form during the last academic year as well as the current year (Primary One students will use the results in the first semester); • (ii) examination scores for three major subjects (Chinese, English, and Mathematics) will be obtained for last year and current year		X	
		• The student’s perception of academic performance in (i) Chinese; (ii) English; (iii) Mathematics; and the overall academic results		X	X
Secondary outcomes					
6. Annual and lifetime prevalence of workplace bullying	Items created by the author	• Two single-item measures of workplace bullying will be completed by working parents. For the annual prevalence, an item such as “Have you ever been bullied at work over the last 12 months?” (Options: Never bullied/Yes, rarely/Yes, sometimes/Yes, Often) will be used. For lifetime prevalence of workplace bullying, an item such as “Have you ever been bullied at work in your life?” (Options: Never bullied/Yes, Rarely/Yes, Sometimes/Yes, Often) will be used.	X		
7. Workplace bullying	Chinese Workplace Bullying Scale (CWBS) [43] with adaptation	• 21 items (e.g., I am assigned a heavy workload.”, “Someone withholds information deliberately to hinder my work performance”, “My efforts at work have been ignored”) • 4-point Likert scale, ranging from 0 “never” to 4 “Often”	X		
8. Mental health	General Health Questionnaire-12 (GHQ-12; Chinese version) [44]	• 12 items, which will be used to measure the severity of the mental problems of parents over the past four weeks • 4-point scale (from 0 “less than usual” to 3 “much more than usual”)	X		

### Data analyses

Statistical analyses will be performed using SPSS. Data will be summarized and presented using appropriate descriptive statistics. The normality of continuous variables will be assessed by skewness and kurtosis statistics and normal probability plot. Skewed variables will be suitably transformed before being entered into statistical analyses. Cronbach’s alpha will be calculated to assess the reliability of the instruments. Pearson correlation analyses will be used to examine the relationships among variables. Cross-sectional data will be used and percentages will be reported for annual and lifetime prevalence of workplace bullying. All statistical tests will be two-tailed and a *p*-value of less than 0.05 will be considered significant.

Structural equation modelling (SEM) will be used to test the model by using M*plus* version 8.0 software package [49]. As missing data can bias results, reduce estimation efficiency, and complicate data analyses, we will estimate the missing data by using Markov Chain Monte Carlo multiple imputation (MCMC-MI), which is more effective than deletion, mean substitution, or simple imputation [50]. The explanatory model will be tested with a structural equation model which reduces the impact of measurement error in the indices and properly models indirect mediation effects [51].

### Time frame for the study

The study started in January 2017 and will last for 30 months. The data collection at Time One has already been completed and data collection for Time Two will commence in September 2018. The whole study will be completed in June 2019 (see Table 3).

**Table 3:** Timeline for data collection

Month	2017												2018												2019					
	1	2	3	4	5	6	7	8	9	10	11	12	13	14	15	16	17	18	19	20	21	22	23	24	25	26	27	28	29	30
Preparation																														
1. Review literature for designing three different questionnaires for parents, teachers and students 2. Liaise and recruit schools for pilot study and main study 3. Prepare consent forms for parents, teachers, and students 4. Apply for ethics approval (4 months)	x	x	x	x																										
Pilot study																														
1. Recruit participants for focus group interviews for operational definition 2. Conduct pilot study in two primary schools 3. Recruit participants to provide feedback on the questionnaire (Organize qualitative interviews for parents, teachers and students) 4. Revise questionnaire 5. Conduct factor analysis, data analysis based on data from pilot study (4 months)				x	x	x	x																							
Main study: Baseline																														
1. Liaise schools for data collection 2. Conduct survey for baseline (18 schools in 18 districts) (8 months)							x	x	x	x	x	x	x	x																
Main study: Follow-up																														
1. Analyze data for Time One 2. Liaise schools for follow-up data collection (5 months)														x	x	x	x	x												
Conduct main study (Follow-up) 1. Collect data for follow up study (8 months)																			x	x	x	x	x	x	x	x				
Completion Phase																														
1. Report writing 2. Analyze data (6 months)																									x	x	x	x	x	x

## Discussion

This study expands the existing literature which focuses on researching the effects of workplace bullying on victims only but has never explored the spillover effects on the victims’ families, in particular, the victims’ children. The study will ascertain whether workplace bullying could lead to serious consequences on children. By exposing the harmful effects of workplace bullying on workers, this study will shed light on the indirect impact of workplace bullying on children’s physical and mental health, externalizing problem behaviors and academic performance via the poor parenting of workers due to chronic stress and/or depressed feelings. Since previous research has indicated that workplace bullying can affect the mental health of workers and since it has been shown that the impaired mental health of parents may have an impact on children development, the findings of this study are therefore expected to highlight the negative consequences of workplace bullying on children and emphasize the urgent need to eliminate bullying in the workplace.

The present study has a number of strengths. First, the study is original because workplace bullying is an under-researched area in Asia and the spillover effects from the workplace environment on parenting has never been researched in the Chinese and Western contexts. In addition, the originality of the study lies in the innovation of its research topic, which covers several areas simultaneously. Workplace bullying belongs to the area of organizational psychology, while child development belongs to the field of developmental psychology. Studying the selected variables simultaneously will therefore enable researchers to better understand the impact of workplace bullying as a growing public health and social issue. Second, the majority of studies on workplace bullying are cross-sectional studies, but the present study will adopt a longitudinal design. It will be interesting to determine whether workplace bullying has any impact on parenting which in turn can affect children health, behavior and academic results over time in the same households. Thirdly, the current study will use multiple informants’ reports, including children, parents and teachers to assess children. Fourthly, this study will yield findings from a large representative sample size because the participants will be recruited from 18 primary schools located in the 18 districts of Hong Kong. Finally, sophisticated data analysis techniques will be used to determine whether parenting mediates the variables of workplace bullying and child’s health, behavior and academic performance. Overall, this study shows significance at different levels, at the individual (i.e., children’s health), familial (parenting), economical and societal levels.

There are some limitations to this study. First, the samples will be Chinese children in Hong Kong, there is a need to replicate the findings in different Chinese communities such as in mainland China and communities in Western contexts. Second, the study is based on self-reported questionnaires so there might be a risk that the participants will only provide socially desirable answers. Therefore, future studies can consider including qualitative interviews to better understand the mechanism of how workplace bullying affects the health of children.

Despite those limitations, to the best of our knowledge, this is a pioneering study in Asia exploring the indirect effects of workplace bullying on family members. The results will be informative and will help the research community and other relevant public services and any other relevant non-governmental organizations (NGOs) make concerted efforts to prevent bullying in the workplace and promote safe and respectful relationships at work which in turn will help improve the mental health of the working population.

## Abbreviations

NGO: Non-governmental organizations
STRN: Student Reference Number
